# Building Community Capacity for Health Promotion in a Hispanic Community

**Published:** 2006-12-15

**Authors:** Marta Sotomayor, Frances Pawlik, Armando Dominguez

**Affiliations:** Marluz Associates. Dr Sotomayor was technical consultant to the Latino Education Project for the REACH 2010 Project when this article was written.; Latino Education Project, REACH 2010 Project, Corpus Christi, Tex; Assistant Director and Health Coordinator, Center on Aging and Health, University of Texas-Pan American, Edinburg, Tex

## Introduction

The Latino Education Project (LEP) is conducting a multilevel Racial and Ethnic Approaches to Community Health (REACH) 2010 diabetes prevention project in Nueces County, one of 12 counties located in the Coastal Bend area of south Texas. Nueces County is characterized by high levels of poverty and diabetes-related complications and disability. The LEP chose a community capacity-building approach to diabetes prevention and health promotion to help midlife and elderly Latinos increase their ability to prevent, control, and manage diabetes and associated disabilities.

In each intervention conducted through the LEP, project workers emphasize the importance of building community health-promotion capacity and recognize the important role that local leaders play in this process. Community-wide health forums, coalitions, and partnership development are key elements in promoting organizational development. These activities increase the social participation necessary for effective community building and problem solving. The use of study circles, or *Ollas del Buen Comer*, are one of the key approaches used to reinforce health-related culture, language-specific needs, and the lifestyle of participants in relation to the environment.

Participation of community lay health educators, *promotores de salud,* is crucial to achieve project goals because of the leadership they provide in their communities. *Promotores* are helpful in one-on-one interactions, and they are crucial in creating environmental changes necessary to reduce the prevalence of risk factors associated with diabetes and other chronic diseases. Some of the tasks required for *promotores* to be effective in this REACH 2010 project are the focus of this discussion.

## Background

In Nueces County, Texas, approximately 33% of Hispanic females and 31.5% of Hispanic males aged 62 years and above have diabetes (1). The prevalence of diabetes in Texas Mexican Americans is as high as 33% in some counties (2). The authors believe that the incidence rate of diabetes among the Hispanic community is greater than reported. Diabetes among many Hispanic residents remains undiagnosed because of their barriers to health care access, including lack of available health care services, lack of knowledge regarding services, lack of familiarity with policies and procedures to obtain health care services, lack of insurance coverage, high cost of health care, lack of transportation to health care facilities, misunderstandings arising from difference in cultural expectations and communication styles, and values and language differences regarding health care (3,4,5).

### Project site

In 2000, the Centers for Disease Control and Prevention (CDC) awarded the LEP a REACH 2010 demonstration grant to focus on health promotion and disease prevention and to address the inordinately high rates of diabetes and its complications in the Coastal Bend area of south Texas. The project is being conducted in Nueces County, which includes the city of Corpus Christi and surrounding smaller rural communities. These rural areas depend on seasonal agriculture-related jobs and have limited resources to sustain a growth economy and needed health and social services.

According to the 2002 U.S. Census (6), 2.6% of the total Texas population resides in the Coastal Bend area. Sixteen percent of residents are aged 60 years and older, and 42% are Hispanic. The population of older Hispanic adults in the Coastal Bend area is fast-growing and diverse and, like other population groups throughout the nation, is living longer (7). 

In Nueces County, 55.8% of the population is Hispanic, and 38.3% is Anglo. In most of the rural communities in the county, the size of the Hispanic population fluctuates, and Hispanic people may at times account for 80% to more than 90% of the total population. Overall, 17.7% of those aged 65 years and older in this area and 28.3% of the Hispanic population aged 65 years and older live below the national poverty level. In the Coastal Bend region, 47.2% of residents did not graduate from high school compared to 24.4% of the population generally in the state of Texas, and 26.9% are high school graduates compared to 24.6% of the population in the state as a whole. In the Coastal Bend region, 18.1% have 12 years of school but no diploma compared to 12.9% of the population in the state, and 34.7% have less than a ninth-grade education compared to 11.5% of the population of the state as a whole (6). The relatively high overall unemployment rate in the area (6.2% in 2002) prompts young Hispanic people to leave the area to seek employment elsewhere.

More than 50% of all deaths in Nueces County can be attributed to chronic diseases, including diabetes and its complications as well as cardiovascular diseases (8,9). Although the county has some health and social service systems with bilingual providers and support staff, public financing for needed services is inadequate. This inadequacy negatively impacts the quality of health care services. The Nueces County area has been medically underserved for a number of years (10).

Sociodemographic and behavioral factors have been associated with increased risk for certain health problems (11). For example, level of education has been correlated with prevalence of health risks such as obesity, lack of physical activity, and cigarette smoking (12). Health-compromising behaviors, such as physical inactivity and poor nutrition, have been clearly linked to increased risk for many chronic diseases (13). Nutrition-related risk factors often work synergistically to adversely affect the health status of older people, and these risk factors may lead to an increased rate of medical complications and result in loss of independence, institutionalization, and higher health care costs for people affected.

Older Hispanic adults in the Nueces County area share socioeconomic characteristics associated with poor health status and experience a high incidence of diabetes-related health problems (e.g., amputations, kidney failure, loss of vision, obesity). Discrimination based on ethnicity, language, culture, and socioeconomic status often isolates and limits older Hispanic adults, and their contact with the Anglo community is generally limited and superficial. Relationships with representatives from outside agencies, particularly those from governmental and health and human service providers, are often characterized by a lack of trust.

Older Hispanic residents share characteristics that enhance a sense of commonality and cohesiveness, and these characteristics result in a strong sense of community. Shared characteristics include ethnicity, language, culture, strong Hispanic (Mexican American) identification, and value for family. Easy access to Spanish-language radio, television, and newspapers is supplemented by easy and frequent travel across the border to Mexico. These ties repeatedly reinforce culture, language, and identity. Most residents, including those who are older, are bilingual in English and Spanish, but the language of preference is Spanish. Through language, residents share a common communication system, formal and informal networks, and personal histories.

### Community

For the past two decades, public health researchers and practitioners have shown increasing interest in community-based approaches to health promotion and disease prevention (14). Evidence suggests that the most effective prevention strategies actively engage the communities they are intended to serve (15). Communities can be mobilized to identify, plan, channel resources, and undertake effective action for health promotion and health-enhancing social change (16). A number of researchers have urged an ecological approach to public health interventions (17) to build on the concept that community is the solution to its problems. There is considerable support for designing community-based interventions to  improve the health behaviors and overall health status of community members (18). These community-based interventions are important because health disparities and the high rate of chronic diseases in minority populations, particularly among those who are poor and lack access to community resources, are not likely to be prevented without them.

*Community* has been defined as a place where identities and meanings are shaped and where values, beliefs, and norms that guide different dimensions of social relationships are rooted (19). Individuals matter to a community, and social ties are valued for their reciprocity and strength. Communities are places where opportunities for behaviors may be offered or limited, and communities may restrict as well as support the growth and health of its members (18). Hispanic people in general, and Mexican American people in particular, value and often define themselves by their strong sense of community.

The LEP chose a community-based strategy to guide its diabetes prevention efforts because of the community-related characteristics of the target population. This strategy supports the idea that communities can create problems, but communities can also provide solutions and a context for health promotion activities focused on prevention and control of diabetes.

### Building community capacity

Building community health promotion capacity is the basic theoretical concept that guides the LEP's community-based interventions to improve health status through the multidimensional REACH 2010 project. Control and management of diabetes are the primary issues addressed by project activities, and project efforts are focused within the context of daily lifestyle activities of the target population group.

The importance of controlling and managing life circumstances, including diabetes-related needs, is integrated into every LEP program activity. Programs are designed to improve the health, economic status, and housing of older Hispanic adults and to address community factors that can affect their ability and that of their families to address systemic problems. The LEP activities focus on the interdependence of people, the importance of mobilizing organizational resources for those most in need, and ways to build social capital (e.g., skills, knowledge, resources) within the Hispanic community for a more equitable distribution of resources.

### The LEP REACH 2010 project

This LEP REACH 2010 project focuses on the development of interventions designed to strengthen the capacity of individuals, social networks, and organizations to create social change and ultimately individual behavior change. To strengthen the capacity of these various systems separately and as a whole, LEP's REACH 2010 project interventions focus on the following themes and messages: 1) building trust among consumers and health care providers; 2) acknowledging the value of cultural, ethnic, and linguistic identities as a strength and catalyst for change; 3) recognizing the importance of social connectedness; 4) improving the quality of ties among community sectors; 5) promoting the importance of individual control over health-related decisions; and 6) seeking to establish organizational relationships and partnerships to mobilize community resources.

The project focuses on three capacity-building strategies: increasing community awareness about the diabetes crisis facing Hispanic communities; solving problems through organizational collaborations and partnerships; and developing community leadership. These strategies work by 1) ensuring participation of community educators or *promotores de salud* in every aspect of project activities; 2) sponsoring community-wide health forums and building coalitions and partnerships; and 3) using study circles, *Ollas del Buen Comer*, to provide health-related knowledge, skill building, and group support.

The first phase of the project emphasized the importance of organizational development as a necessary antecedent to community participation, and action focused on two sets of activities: strengthening the LEP as a community-based, nonprofit, advocate organization that convenes, influences, and mobilizes other organizations; and promoting organizational collaboration and problem solving through coalition and partnership building to promote community ownership of the diabetes-related crisis.

The present phase of the project emphasizes the importance of skill development in building community capacity to promote social and individual change and in building knowledge and skill development of *promotores*. The development of a data system to track indicators of the effectiveness of *promotores* is part of this process.

## Program Planning

### Coalition and partnership building

As part of building community capacity, the LEP REACH 2010 project began with the premise that health promotion programs must demonstrate a capacity and willingness to allow community priorities to guide program development and services. This premise required the inclusion of other community organizational stakeholders at the beginning rather than as an afterthought in the design and implementation of this prevention project. The LEP began by forming an action-oriented partnership that included elected officials, older adults, and representatives of community groups and agencies (e.g., hospitals, educational institutions, nutrition centers). The initial purpose of the coalition was to establish a network of organizations to examine local resources and eventually to develop a community plan to reduce community-level barriers to health care services.

Coalition members involve their professional networks to expand the scope of the coalition, identify project gaps, suggest new programs and approaches, and locate funding sources. The coalition meets regularly, reviews REACH 2010 interventions, and makes suggestions to ensure that LEP activities represent the interests and well-being of the community. To increase the involvement of coalition members, the LEP offers a number of opportunities to participate in its various diabetes interventions.

### Focus groups

The Center on Aging and Health (CoAH) at the University of Texas-Pan American conducted a series of focus groups in 2001, a year before the REACH 2010 project began, to gain insights from the analysis of  focus group data to help LEP staff design effective health prevention programs and to develop efficient strategies for reaching out to midlife and older Latino populations (20).

A total of 96 Mexican American adults aged 65 years and older participated in eight focus groups that took place in settings that ensured diversity of group composition. All groups were conducted in Spanish and were videotaped. Three staff members independently watched each videotape and met later to discuss their notes and identify the major points raised by the focus groups. The principal investigator watched all videotapes a second time and prepared the findings for analysis. All three staff members met again to review the prepared findings and arrived at a consensus on the major points that were independently observed regarding overall diabetes-related knowledge and the needs of focus group participants.

Key findings were that focus group participants 1) had limited knowledge about diabetes and cardiovascular disease and were unable to identify symptoms early and at the most appropriate time for effective treatment and control; 2) were aware that proper diet and exercise contributed to the management and control of diabetes and cardiovascular disease; 3) were aware that proper diet and exercise were powerful preventive mechanisms that would dramatically lower their risk of ever having the diseases and that this awareness among most group participants who indicated they had one or both conditions came late in life and only after they were diagnosed with the disease; and 4) expressed a desire to increase their knowledge and understanding of their diseases as a means of preventing serious complications.

All participants expressed problems adhering to a healthy diet and a moderate exercise schedule and had trouble associating any form of exercise with pleasure. Participants who had been or were avid dancers did not see dancing as a form of exercise. Most admitted that they should walk more than they actually did, but few gave any concrete indication as to what kind of strategy would allow them to do so on a regular basis. Most participants found foods high in carbohydrates and calories to be pleasurable, particularly the typical Mex-Tex cuisine, and expressed little motivation to change to healthier foods.

Most participants did not see access to health care as problematic or difficult. All reported that they had Medicare, and most had seen a doctor during the past 6 months. Most were satisfied with their doctors despite the brevity of their visits. Participants acknowledged this brevity prevented any opportunity for meaningful patient–doctor dialogue that might help them better understand their health conditions. No participants expressed a problem paying for medical bills, but many expressed their belief that they were overmedicated and had no opportunity to discuss this issue with their doctor or other health care provider. Most participants noted that transportation was a problem since they depended on either a child or a senior-center van to take them to medical appointments.

The findings of the focus groups highlighted a number of themes that were incorporated in the different REACH 2010 interventions, including

- increasing older Hispanic adults' knowledge of diabetes and cardiovascular diseases;
- changing participants' attitudes and behaviors to diminish risk for diabetes and cardiovascular diseases;
- identifying prevention strategies that work and strategies that do not work with this population group;
- examining barriers associated with the present health care system that limit interactions between patient and health care provider; and
- developing outreach programs for local neighborhoods.

## Interventions

### Building human capital and leadership: *promotores de salud*

Involvement of *promotores de salud* (e.g., health promoters, health facilitators, health supporters, lay health educators, community health workers) is a fundamental dimension of the community-building framework because these workers share a deep understanding of beliefs, perceptions, and salient concerns with the populations and communities with which they work. They help fill gaps in health care and human services for medically underserved and resource-poor communities and have been particularly effective in reaching rural, minority, and other socioeconomically disadvantaged populations (21).

Most programs that use lay health educators emphasize educators' ability to help people access health and human services. The community-building approach the LEP uses has *promotores de salud* as essential human capital to perform leadership roles that result in social change as well as individual behavior change. Primary targets for interventions include organizational systems, communication channels, and small groups in which *promotores* can play active roles in initiating changes that are crucial to health promotion and disease prevention for both communities and individuals (22).

The 2001 National Community Health Advisor Study conducted by the American Public Health Association identified seven core roles performed by community health workers: 1) providing cultural mediation between communities and health and social service systems; 2) providing culturally appropriate health education and information; 3) ensuring that people get the services they need; 4) providing informal counseling and social support; 5) advocating for individual and community needs; 6) administering health screening tests; and 7) building individual and community health care capacity (23, 24).

In the LEP's REACH 2010 project, *promotores* are expected to help change environmental factors to reduce individual risk for certain diseases. Rather than using *promotores* in their usual roles (e.g., case managers, coordinators of services), the LEP begins with the desired outcomes of each intervention, determines the knowledge and skills required to accomplish the intervention, and then seeks *promotores* who have the necessary knowledge and skills. *Promotores* are expected to be leaders in creating social and economic conditions that help individuals control and manage their diseases. Effective leadership requires communication, analytical skills, and teaching skills as well as abilities in coaching, creating a vision, building trust, teamwork, reflection, learning, and partnering (23). *Promotores* can mobilize resources and are valuable tools in building a community's capacity to help those in need.

### Establishing community health forums

The LEP sponsors four community-wide health forums each year. These forums are designed to raise awareness of the many risk factors for type 2 diabetes mellitus among older Hispanic adults and their families and to demonstrate methods of managing and controlling these risk factors. Goals of these forums are to improve community capacity to identify and treat residents with diabetes, increase community participation in health-related activities, and provide opportunities for local leaders to address diabetes-related issues at the community level. Information about causes and control of diabetes provided at the forums is appropriate to the culture, language, age, and education level of the audience.

Community forums are cosponsored by local agencies that partner with the LEP's REACH 2010 project and are conducted by volunteers. Student nurses from a local community college provide basic screening tests, the local HMO (Humana) demonstrates an age-appropriate exercise program, and local physicians participate in presentations on diabetes and its complications. Forum participants are offered screening tests for hypertension, high cholesterol, and diabetes as well as foot and eye examinations. Local agencies, such as home health care agencies, nursing homes, and senior centers, advertise their services, distribute informational materials, and give participating seniors an opportunity to learn about services available in the community and how to access them.

Community forums bring together representatives of community organizations, health care and social service professionals, advocates for seniors, elected officials, representatives of public and private health care and human service agencies, and seniors. Presentations are bilingual, interactive to encourage maximum audience participation, and use cultural symbols, communication styles, and visual materials appropriate for older citizens who may be illiterate in English, Spanish, or both.

Assessments about the effectiveness of these community forums are based on the number of participants at each event, who the partners or cosponsors were, and which organizations or individuals contributed concrete resources. Examples of contributions include 1) providing the site for the event (usually contributed by a church, city, or community center); 2) providing basic screening tests; 3) providing free media announcements about the event; and 4) providing meals and gifts for participants. Assessments are also based on participant feedback about the value of presentations in meeting individual needs.

Conversations with participating seniors indicate an increase in knowledge about diabetes and its risk factors as a result of health forum attendance. Because a number of seniors attend these community events repeatedly, LEP staff, in collaboration with the CoAH at the University of Texas-Pan American (a project partner), have begun to develop evaluation tools to assess diabetes-related knowledge gained by participants over time and to determine which topic, style of presentation, and individual presenters are most effective in promoting healthy lifestyle and behavior changes.

*Promotores de salud* assume primary responsibility for outreach to various community sectors represented at community forums. *Promotores* design a community-wide media campaign and participate in selecting messages and a communication style that are appealing to older Hispanic adults. *Comentarios* (exchange of ideas), a program sponsored by a local Spanish-language radio station, interviews people at event sites and encourages exchanges between seniors, agency representatives, local leaders, health professionals, and *promotores.* A quarterly newsletter, *Nuestra Salud*, is published to inform the community about these events and to provide information about diabetes and its management to more than 1500 organizations and individuals.

The success of these community events reflects the importance of involving community leaders who understand the community and its needs. Forums are bilingual and reflect community culture. Participants are shown how to mobilize community resources so that residents can access resources easily. These events are influential, visible, and credible, and they bring together participants from many organizations and community sectors.

### Establishing study circles: *Ollas del Buen Comer*

In addition to sponsoring community events designed to reach a broad segment of the community, the LEP also sponsors a series of small group sessions called *Ollas del Buen Comer* (Skillets for Healthful Eating) to focus attention on individual participants within their own cultural context. These time-limited sessions have a dual function: to teach participants diabetes-related self-care practices (e.g., nutrition, physical activity) and to provide them with group support by using cultural identification as a tool for behavior change.

In these study circles, participants are encouraged to think critically about cultural assumptions underlying their health-related ideas and actions. They have opportunities to examine their lifestyle behaviors and beliefs and how these affect their health and nutrition. In small interactive sessions, participants consider alternative ways of thinking and behaving and look for lessons from past actions. They are encouraged to develop self-awareness and to view errors and failures as resources for learning instead of excuses for not acting or as reasons to blame themselves.

*Ollas del Buen Comer* have been particularly effective in helping participants examine barriers to making diet and exercise behavior changes. Participants examine their financial limitations, family customs and habits, and attitudes (e.g., fatalism about diabetes, skepticism about the benefits of prevention). They discuss other barriers such as a lack of transportation to reach stores where there are lower food prices and dental problems that prevent them from consuming certain foods. Presenters and participants identify what nutrition education is needed and how nutrition information can best be presented. *Promotores* are particularly suited to interact with their peers in a study-circle format because of their culturally appropriate manner and knowledge of language, culture, and common historical roots.

*Ollas del Buen Comer* sessions include food-preparation demonstrations with culturally appropriate, inexpensive, and accessible foods, such as *nopalitos* or springtime fresh cactus pods. Sessions include demonstrations of age-appropriate physical activities (e.g., stretch exercises, walking, dancing), Heart Smart sessions on cardiovascular disease adapted from American Heart Association literature, and presentations on diabetes-related issues adapted from American Diabetes Association materials about proper foot and eye care and explanations about the differences between type 1 and type 2 diabetes mellitus.

*Promotores* are facilitators and mediators. They help participants navigate health care and human service systems, clarify and interpret community resources, and encourage those who need them to use appropriate services. *Promotores* help participants reflect and think through their problems and connect with health care and social service providers as they seek solutions. They lead participants through necessary steps for lifestyle changes and raise individual awareness about potential barriers to such changes.

The LEP has developed a computerized data management system to help *promotores* track activities and outcomes and identify indicators of effectiveness. With assistance from its university partner, the LEP is developing the necessary tools to determine outcomes.

## The Importance of the REACH 2010 Program

The high rates of diabetes and diabetes-related complications among elderly Hispanic populations such as those in Nueces County, Texas, need to be understood in terms of the environment in which they exist. This understanding cannot be attained quickly nor can the problems associated with this health disparity be solved soon. Problems take years, and sometimes generations, to develop, and solutions require ongoing analyses and application of resources over time.

Community-based nonprofit organizations can play an important role in resolving the diabetes crisis faced by Hispanic communities through effective interventions. Long-term financial support provided by the REACH 2010 project has significantly diminished the traditional funding instability that has often undermined the ability of these organizations to fully achieve their goals. The REACH 2010 project plays an equally important role by focusing on community-wide, comprehensive, multilayered strategies with which to identify, develop, and test knowledge and skills critical for effective community building in minority communities.

The next step for the LEP is to focus on finding resources to document the contributions that *promotores* make to promote healthy communities. The LEP will continue its involvement in a statewide effort to develop effective training curricula with defined core roles and competencies for *promotores*. More work is needed to determine how the roles of *promotores* are different from those of other service providers in building community capacity and leadership. Challenges that the LEP REACH 2010 project needs to address include selecting a process for standardized baseline data collection and developing research methods that capture the subtleties of *promotores' *strategies, such as their approach to differing consumer cultures and communities. Observations thus far indicate that *promotores* contribute to improved health status in Hispanic communities by encouraging use of appropriate health services and by implementing prevention and health promotion services.
